# Development of a predictive model for predicting disability after optic neuritis: a secondary analysis of the Optic Neuritis Treatment Trial

**DOI:** 10.3389/fneur.2023.1326261

**Published:** 2024-01-08

**Authors:** Siqian Wei, Yi Du, Meifeng Luo, Ruitong Song

**Affiliations:** Department of Ophthalmology, The First Affiliated Hospital of Guangxi Medical University, Nanning, China

**Keywords:** multiple sclerosis-related optic neuritis, neurological disability prediction, EDSS, prediction model, personalized medicine

## Abstract

**Objective:**

The present study aimed to develop a prediction model for predicting developing debilities after optic neuritis.

**Methods:**

The data for this research was obtained from the Optic Neuritis Treatment Trial (ONTT). The predictive model was built based on a Cox proportional hazards regression model. Model performance was assessed using Harrell’s C-index for discrimination, calibration plots for calibration, and stratification of patients into low-risk and high-risk groups for utility evaluation.

**Results:**

A total of 416 patients participated. Among them, 101 patients (24.3%) experienced disability, which was defined as achieving or surpassing a score of 3 on the expanded disability status scale. The median follow-up duration was 15.5 years (interquartile range, 7.0 to 16.8). Two predictors in the final predictive model included the classification of multiple sclerosis at baseline and the condition of the optic disk in the affected eye at baseline. Upon incorporating these two factors into the model, the model’s C-index stood at 0.71 (95% CI, 0.66–0.76, with an optimism of 0.005) with a favorable alignment with the calibration curve. By utilizing this model, the ONTT cohort can be categorized into two risk categories, each having distinct rates of disability development within a 15-year timeframe (high-risk group, 41% [95% CI, 31–49%] and low-risk group, 13% [95% CI, 8.4–17%]; log-rank *p*-value of <0.001).

**Conclusion:**

This predictive model has the potential to assist physicians in identifying individuals at a heightened risk of experiencing disability following optic neuritis, enabling timely intervention and treatment.

## Introduction

Optic neuritis, a demyelinating inflammation of the optic nerve, affects 3–5 people per 100,000 people annually worldwide (1). The Optic Neuritis Treatment Trial (ONTT) found that most patients with optic neuritis in the study had a connection with multiple sclerosis (MS). This condition can cause substantial visual impairment, impacting patients’ quality of life. These visual or physical impairments due to MS could potentially evolve into permanent disabilities (2). During the 15-year follow-up of the ONTT, the Optic Neuritis Study Group observed that patients with optic neuritis generally had good visual acuity outcomes throughout the long-run follow-up. Although 61% of these patients regained their vision with a visual acuity of 20/20 or better in both eyes, some of them continued to experience persistent visual impairment in other aspects of visual function, including the visual field, color vision, and contrast sensitivity (3–6). According to the National Eye Institute Visual Function Questionnaire scores, they still perceived their visual function to be poorer than a disease-free population, especially those who had a visual or physical disability due to MS, whose vision experience was worse than healthy people (3).

A customized treatment strategy must start with the early detection of patients who are at risk for disability. Previous studies have found that factors including cortical fractal dimension, lesion on magnetic resonance imaging (MRI), and retinal measurements are associated with disability after optic neuritis (7–14). However, single factors, such as current clinical and paraclinical measures, are not sufficient to reliably predict the risk of disability. Therefore, there is a need for developing risk prediction models that can incorporate multiple factors to improve the accuracy and precision of the prognosis.

Our study aimed to develop a predictive model of disability using the ONTT cohort to identify patients with optic neuritis likely to experience disability, identify risk factors predicting significant visual or motor impairment, and develop a predictive model estimating the likelihood of disability over 10- and 15-year timeframes. Our study aimed to provide more accurate and reliable model to identify those at a high risk of disability and to guide treatment decisions.

## Method

We employed a publicly available dataset from the ONTT, accessed through the website http://lons.jaeb.org/. The design of the ONTT, a randomized controlled trial, has been described in previous studies (15–19). In short, the ONTT was conducted to determine the effectiveness of corticosteroids for acute unilateral optic neuritis and the proportion of patients who later developed MS. The ONTT enrolled participants between the ages of 18 and 46 years as long as they had experienced visual symptoms for no more than 8 days.

The ONTT randomly assigned patients among three treatment groups: the high-dose intravenous methylprednisolone group (1,000 mg every day for 3 days), the low-dose oral prednisone group (1 mg per kg per day for 14 days), and the oral placebo group. At baseline, 6 months, and 12 months, as well as annually for 5 years after enrollment, standardized ophthalmological and neurological examinations were performed, followed by evaluations at 10 and 15 years. The ONTT conducted a follow-up on the visual prognosis of these patients and recorded the patients’ expanded disability status scale (EDSS) scores (20).

In the ONTT, the diagnosis of MS was made based on the diagnostic criteria for MS proposed by Poser et al. (21). MS was classified into four categories: none, possible, probable, and definite.

In this analysis, patients were divided into those with no or negligible disability (an EDSS score less than 3) and those demonstrating clinically notable disability (an EDSS score equal to or exceeding 3). The patients who had no EDSS score during follow-up were excluded from this analysis.

The ONTT strictly adhered to the principles outlined in the Declaration of Helsinki and received approval from the institutional review boards at each of the implementing institutions. All participants in the ONTT willingly provided informed consent when enrolling in the trial.

### Statistical analysis

Categorical variables were denoted by precise numerical values and corresponding ratios, whereas continuous variables were indicated by average values accompanied by standard deviations or medians along with interquartile ranges, depending on whether the distribution of the data is normal or non-normal. The Kaplan–Meier method was used to estimate the probability of developing a disability in the study population, and the test of log-rank was employed to contrast variations between groups in disability occurrence and development rates. Variables with more than 50% missing values were removed before starting modeling. In the model, categories of similarity coefficients were combined (for example, possible and probable MS were combined into a single category). We used all available cases of the ONTT for modeling; therefore, we did not consider the sample size calculation.

The candidate variables in the model were selected based on clinical relevance, prior research (22), and data availability. These variables included age, sex, number of lesions on MRI, treatment group (intravenous, placebo, and prednisone), visual acuity of the affected eye, the baseline condition of the optic disk in the affected eye, pain status of the affected eye, the presence or absence of the optic disk or peripapillary hemorrhage in the affected eye, whether the person lived in the north for 10 or more years of the first 15 years of life (with ‘north’ defined as states predominantly located above latitude 40° north), the baseline classification of MS (none, possible, probable, and definite), and prior neurologic symptoms.

Analysis of the missing data revealed a random distribution of missing information related to brain lesions, as detected on MRI scans. Using the *mice* package in R, the missing data were estimated as multivariate estimates by chaining equations. Five imputed datasets were generated and modeled separately, and the estimated values were pooled according to Rubin’s rules (23).

We employed the Cox proportional hazards regression model to evaluate the association between potential predictors and the development of disability. The results are presented as a 95% confidence interval (CI) using the hazard ratio (HR). Based on the Akaike Information Criterion, we used a backward stepwise selection procedure to select potential predictors. Finally, the baseline classification of MS and the baseline condition of the optic disk in the affected eye were integrated into the multivariable Cox proportional hazards regression model.

Using the *rms* and *Shiny* packages, a nomogram and an online calculator were developed to forecast the likelihood of disability development. Harrell’s C-index (24) and calibration curves were utilized to evaluate the effectiveness of the predictive model. The cumulative rates of disability development between the high- and low-risk groups were assessed using the Kaplan–Meier survival curves, which were stratified based on individual predicted total scores. A significance level of *p* of <0.05 was considered for all two-sided tests. All data were analyzed using R 4.0.5 (25).

## Results

### Demographic and clinical features

**Figure 1:**
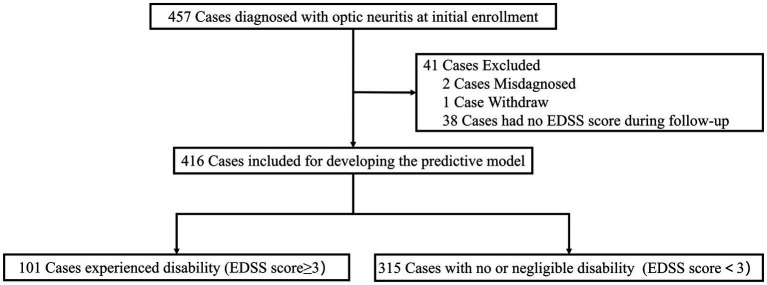
Study flowchart.

In the initial ONTT, a total of 457 cases were enrolled for investigation. Two patients were initially misdiagnosed with optic neuritis, and one patient withdrew before completing the baseline neurological examination. Additionally, 38 cases were excluded from our analysis due to unavailable EDSS scores. Therefore, 416 cases were included for building the predictive model (Figure 1).

At baseline, the age of the 416 cases was 32 on average (standard deviations, 6.8 years), with women comprising 79% (327/416) of the cohort. A significant majority of the participants, namely 87% (362/416), identified as white. Out of the 416 cases, 33 cases (7.9%) were diagnosed with definite MS at the time of their initial enrollment. Additionally, 68 cases (16.3%) were classified as possible MS and 24 cases (5.8%) as probable MS. Moreover, 32% (133/416) of the cases were assigned to high-dose intravenous methylprednisolone treatment, 35% (147/416) to low-dose oral prednisone, and 33% (136/416) to the placebo group. Among the 379 participants with baseline MRI data available, 47% (193/379) showed at least one brain lesion in their baseline MRI. Notably, 91% of the participants exhibited eye pain, while optic disk edema was observed in 36% of the participants. A significant proportion of patients, namely 58%, had lived in the north for 10 or more years of the first 15 years of their lives. Neurological impairment was predominantly mild among the participants. A small proportion of patients (16.1%, 67) experienced moderate disability (EDSS score of 3.0 to 5.5) during the follow-up period and an even smaller group (8.2%, 34) exhibited severe disability (EDSS≥6.0). The probability of developing a disability at 15 years stood at 22% (95% confidence interval [CI], 17–26%).

### Predictors in the predictive model

**Figure 2:**
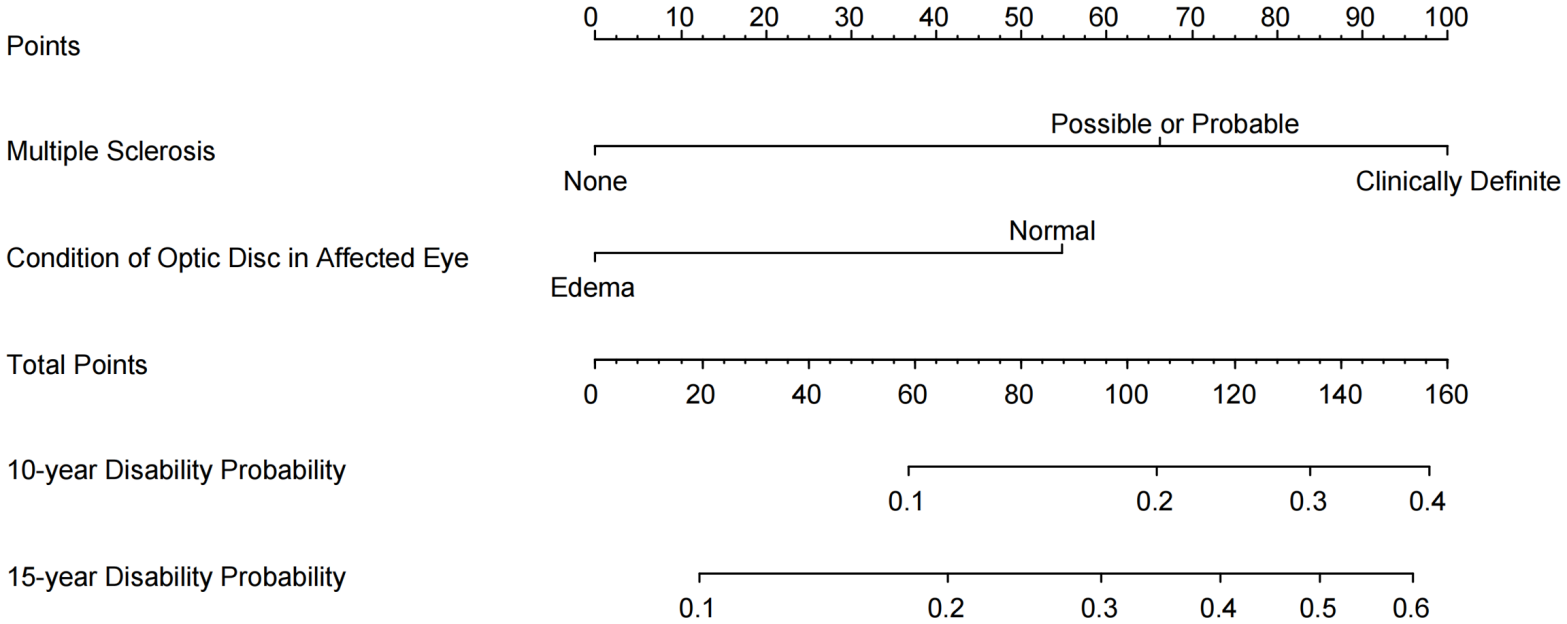
Nomogram for predicting the probability of disability after optic neuritis. An online user-friendly calculator of disability probability is available at https://drduyi.shinyapps.io/edss.

Potential predictors were assessed using the univariate Cox proportional hazards regression model (Table 1). Our novel prognostic model incorporated two factors, including the baseline classification of MS (none, possible, probable, and definite), and the baseline condition of the optic disk in the affected eye (Table 1). In the final model, for MS classification, the HR was 2.92 (95% CI, 1.89, 4.52) for “Possible or Probable” and 5.04 (95% CI, 2.92, 8.68) for “Definite,” with “None” serving as the baseline (HR: 1). Regarding optic disk edema, the HR was 2.42 (95% CI, 1.47, 4.00) for “Normal,” with “Edema” as the baseline (HR: 1). These two factors were explicitly identified as the exclusive predictive factors retained in our final model. The nomogram illustrating the model for predicting the probability of disability conversion after optic neuritis is presented in Figure 2. An online calculator for assessing disability probability can be accessed at https://drduyi.shinyapps.io/edss.

**Table 1:** Cox proportional hazards regression model showing the association of different variables with the disability after optic neuritis.

Baseline characteristics	Univariable		Multivariable (final model)	
	HR (95%CI)	*p*-value	HR (95%CI)	*p*-value
Factors selected				
**Classification of multiple sclerosis**				
None	1 [Reference]	–	1 [Reference]	–
Possible or probable	2.98 (1.92, 4.61)	<0.001	2.92 (1.89, 4.52)	<0.001
Definite	5.49 (3.19, 9.45)	<0.001	5.04 (2.92, 8.68)	<0.001
**Optic disk edema in the affected eye**				
Edema	1 [Reference]	–	1 [Reference]	–
Normal	2.62 (1.59, 4.32)	<0.001	2.42 (1.47, 4.00)	0.001
**Factors not selected**				
**Age**	1.01 (0.98, 1.04)	0.620	–	–
**Sex**				
Female	1 [Reference]	–	–	–
Male	0.89 (0.54, 1.47)	0.659	–	–
**Brain lesions on MRI**				
No lesions	1 [Reference]	–	–	–
1–2 Lesions	0.97 (0.52, 1.81)	0.916	–	–
≥3 Lesions	2.60 (1.67, 4.05)	<0.001		
**Treatment group**				
Placebo	1 [Reference]	–		
Intravenous	1.13 (0.67, 1.89)	0.652		
Prednisone	1.54 (0.96, 2.48)	0.073		
**Visual acuity in the affected eye (logMAR units)**	1.29 (0.97, 1.73)	0.086		
**Ocular pain in the affected eye**				
No	1 [Reference]	-	-	-
Yes	0.91 (0.47, 1.75)	0.782	-	-
**Disk or peripapillary hemorrhage in affected eye**				
No	1 [Reference]	–	–	–
Yes	0.57 (0.18, 1.78)	0.331	–	–
**Lived in the north for 10 or more of the first 15 years of life**				
No	1 [Reference]	–	–	–
Yes	0.80 (0.54, 1.18)	0.252	–	–
**Prior neurologic symptoms**				
No	1 [Reference]		–	–
Yes	1.89 (1.18, 3.01)	0.008	–	–

HR, hazard ratio; LogMAR, logarithm of the minimum angle of resolution.

### Predictive model performance

**Figure 3:**
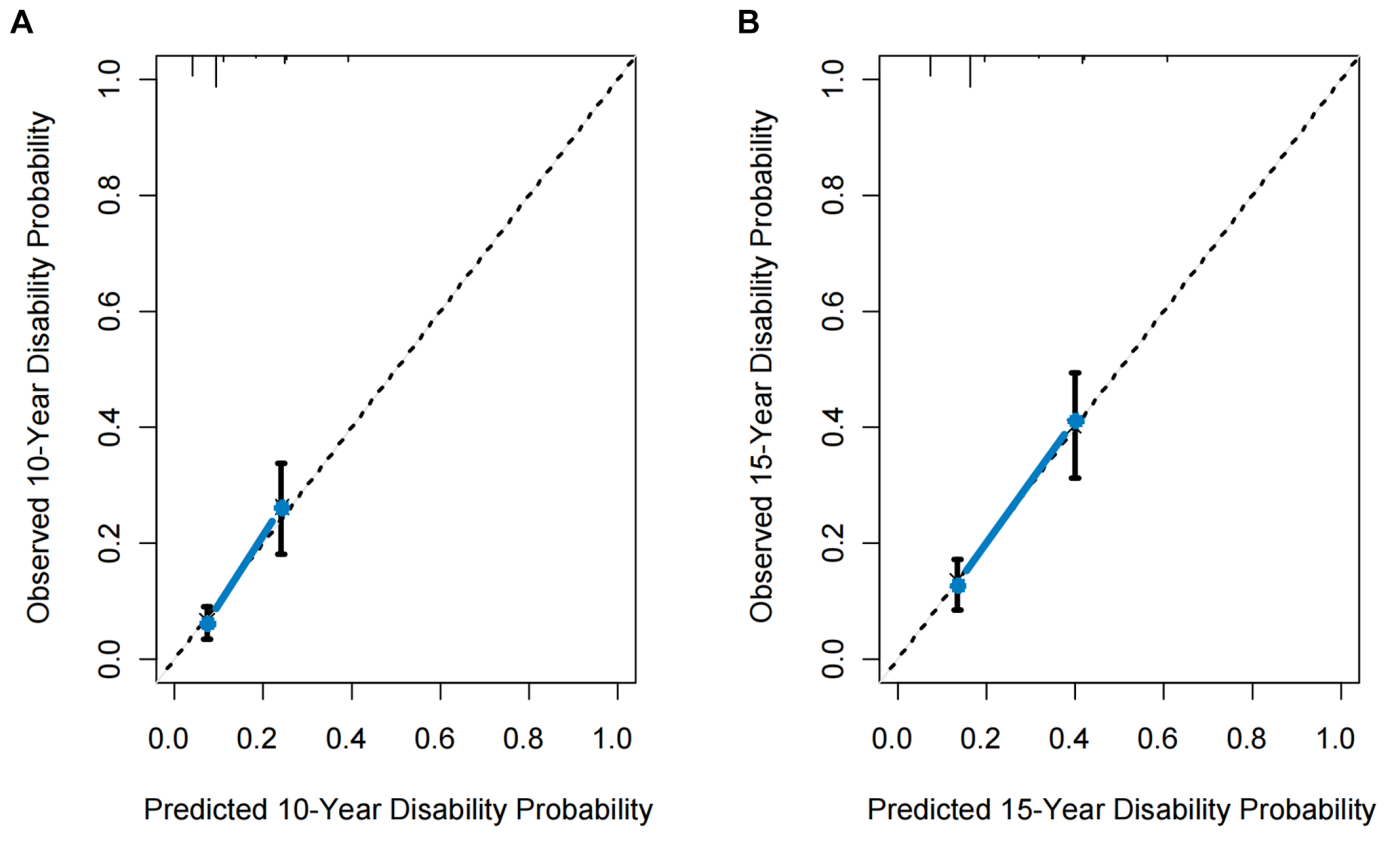
Calibration curves for the proposed predictive model. **(A)** 10-year calibration curve. **(B)** 15-year calibration curve. The dotted line represents the ideal fit. Circles represent model-predicted probabilities, and crosses represent the bootstrap-corrected estimates. Error bars indicate 95% confidence intervals.

**Figure 4:**
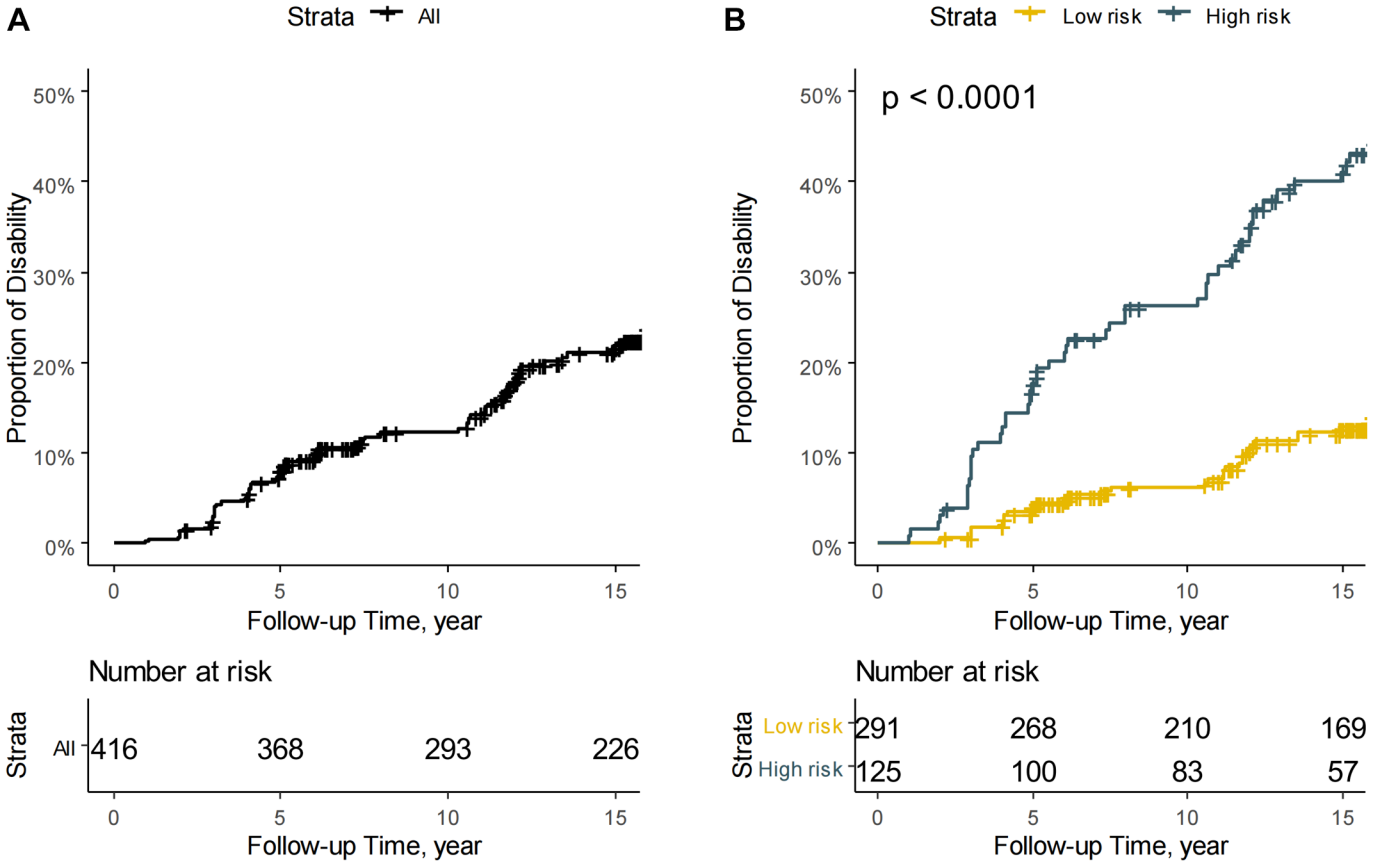
Kaplan–Meier survival curves. **(A)** Kaplan–Meier survival curve for the total ONTT cohort. **(B)** Kaplan–Meier survival curves for the ONTT cohort divided into two risk groups based on the proposed predictive model, showing different probabilities of disability.

The model’s C-index was 0.71 (95% confidence interval: 0.66–0.76, with an optimism of 0.005). The calibration plots for the predictive model assessing the development of disability demonstrate excellent performance (Figure 3). Using the model, the ONTT patient cohort was divided into two risk groups based on the likelihood of developing a disability within a 15-year period. These risk groups’ rates of being disabled differed noticeably: the high-risk group had a rate of 41% (95% CI, 31–49%) and the low-risk group had a rate of 13% (95% CI, 8.4–17%) (log-rank *p* < 0.001) (Figure 4).

## Discussion

Using our novel predictive model, we stratified the ONTT cohort into low- and high-risk groups for disability development after optic neuritis, which allows the patient to understand the potential for future disability and facilitates the development of treatment strategies with the doctor.

It is essential to identify patients at risk for disability as early as possible to develop personalized management strategies. A recent study by Martinez-Lapiscina et al. (26) demonstrated that baseline retinal layer measurements may predict disability worsening over the subsequent 5 years. Another study has shown that the macula may be a retinal region of particular interest when assessing the burden of neurodegeneration, as MS patients with significantly thinner macula showed higher levels of disability (7).

Additionally, research highlights that factors such as male gender, older age at symptom onset (27), and the presence of initial symptoms at the onset of MS (28) are significantly linked to a less favorable prognosis (29). The research on the natural history of MS has found that, compared to the slow onset of motor dysfunction, limb ataxia, or balance impairment that suggests cerebellar involvement symptoms in MS, the presence of optic neuritis at the onset of MS is associated with favorable outcomes in MS (30). In the case of MS-related optic neuritis, disability usually develops during the later stages of MS, which is because MS affects various regions of the brain and spinal cord, leading to a deceleration or blockage in nerve signal transmission between neurons. These effects result in neurological symptoms that may ultimately lead to a diminished quality of life and disability (31). Prior studies have shown that, after 10 years, two-thirds or more of patients with MS-related optic neuritis have a minor disability; however, after 15 years, up to half of the people may need assistance walking (32–34). In our finding, we observed that patients with optic neuritis who had clinically definite MS at baseline had a higher risk of developing disability compared to those who were not diagnosed with MS.

In the ONTT, high-dose intravenous methylprednisolone may reduce the risk of MS occurrence (22), and in theory, this decrease may also lower the risk of disability. However, our analysis did not observe such an occurrence, so further research is needed to investigate the actual impact of corticosteroid therapy on disability. The Optic Neuritis Study Group has established that patients without a swollen optic disk have a higher likelihood of developing MS (35). Similarly, our study has revealed that patients with MS-related optic neuritis who exhibit non-swollen optic disks at baseline are more predisposed to evolving into disability.

Furthermore, various studies shed light on the relationship between treatment measures and the likelihood of disability development. Disease-modifying therapies (DMTs) have shown the ability to delay the progression from optic neuritis to MS (36). An analysis of the impact of interferon therapy on the evolution of disability after optic neuritis reveals that individuals with MS who experience one or more relapses within the first 2 years of interferon therapy tend to face earlier and more persistent disability (37). Motamed et al. (38) found that interferon-beta-1a (Rebif) DMT may effectively halt the development of impairment.

With the advent of high-efficacy DMTs, there have been studies conducting long-term follow-ups on various high-efficacy DMTs, such as natalizumab (39). Among individuals with relapsing–remitting MS, those treated with ocrelizumab exhibited reduced frequencies of relapses, disability advancement, and MRI activity when juxtaposed with patients treated with interferon-beta (40). While interferon was initially a pivotal DMT for MS, the emergence of new and highly effective DMTs has led to a shift in preferences toward these high-efficacy options. It is crucial for individuals with MS to receive appropriate medical care and support to help manage their symptoms and improve their overall functioning. Preliminary findings from the UK risk-sharing scheme have highlighted the potential advantages of DMT in mitigating the development of disability (41).

Different perspectives exist on the predictive relationship between baseline MRI scans and disability outcomes. Swanton et al. found that the presence and quantity of spinal cord lesions at baseline and new T2 lesions during follow-up were significant independent predictors of high disability rates in the whole clinically isolated syndrome cohort (42). Fisniku et al. (43) discovered that initial brain MRI findings predicted the development of clinically definite. Lesion volume and its early changes are correlated with disability after 20 years. Their findings suggest that the existence and number of lesions observed at baseline brain MRI may serve as predictive factors for disability in MS-related optic neuritis cases. However, Beck et al. (44) found that, in the ONTT cohort, there was no significant correlation between the level of disability after 10 years and the presence or number of lesions in the initial brain MRI scans. The discordance observed between these studies that focus on clinically isolated syndrome and the conclusions of Beck et al.’s study may stem from a variety of factors. These factors include genetic and environmental influences, as well as disparities in the timing of the research. Furthermore, variations in the patient cohorts studied at different time points could potentially have had an impact on the results. In our study, guided by the Akaike Information Criterion, relevant MRI parameters were not included.

## Limitations

While our study successfully achieves its objective of developing prognostic model for disability prediction, several limitations warrant consideration. First, the widespread applicability of the research outcomes may be constrained due to a lack of external validation data. Second, in the serotype examination of 177 ONTT participants, no cases of aquaporin-4-IgG+ ON were found, and only a negligible proportion (1.7%) of participants presented myelin oligodendrocyte glycoprotein-IgG+ ON (45), rendering our model inapplicable to patients with either condition. Third, our model does not take into account other potential factors that could affect the prognosis of disability, such as MRI-related parameters, patient lifestyles, health conditions, or data from OCT monitoring. Finally, given that our data originates from a comparatively early period, our model relied on earlier MS diagnostic criteria that differed from the current McDonald criteria. In practical application, we suggest incorporating a re-diagnosis step based on the old criteria into the modeling process before utilizing our model, ensuring its usability under the new standards. Despite these limitations, internal validation indicates that our proposed predictive model maintains robust discrimination and calibration.

## Conclusion

Using the dataset from the ONTT, we have developed a predictive model for predicting disability after optic neuritis. Our prediction model can provide the absolute risk likelihood of developing disability after optic neuritis, which facilitates the co-development of disease management strategies by physicians and patients.

## Data availability statement

The raw data supporting the conclusions of this article will be made available by the authors, without undue reservation.

## Ethics statement

The studies involving humans were approved by the institutional review board at each of The Optic Neuritis Study Group’s execution centers. The studies were conducted in accordance with the local legislation and institutional requirements. The participants provided their written informed consent to participate in this study.

## Author contributions

SW: Data curation. Formal analysis, Investigation, Methodology, Writing – original draft, Conceptualization, Writing – review & editing. YD: Writing – review & editing, Conceptualization, Data curation, Formal analysis, Investigation, Methodology, Writing – original draft. ML: Writing – review & editing. RS: Writing – review & editing.

## References

[1] Asseyer S Asgari N Bennett J Bialer O Blanco Y Bosello F. The acute optic neuritis network (ACON): study protocol of a non-interventional prospective multicenter study on diagnosis and treatment of acute optic neuritis. Front Neurol. (2023) 14:1102353. doi: 10.3389/fneur.2023.1102353, PMID: 36908609 PMC9998999

[2] Dobson R Giovannoni G. Multiple sclerosis—a review. Eur J Neurol. (2019) 26:27–40. doi: 10.1111/ene.13819 30300457

[3] Optic Neuritis Study G. Visual function 15 years after optic neuritis: a final follow-up report from the optic neuritis treatment trial. Ophthalmology. (2008) 115:1079–1082.e5. doi: 10.1016/j.ophtha.2007.08.004 17976727

[4] Cole SR Beck RW Moke PS Gal RL Long DT. The National eye Institute visual function questionnaire: experience of the ONTT. Optic neuritis treatment trial. Invest Ophthalmol Vis Sci. (2000) 41:1017–21. PMID: 10752936

[5] Sanders EA Volkers AC van der Poel JC van Lith GH. Estimation of visual function after optic neuritis: a comparison of clinical tests. Br J Ophthalmol. (1986) 70:918–24. doi: 10.1136/bjo.70.12.918, PMID: 3801369 PMC1040861

[6] Fleishman JA Beck RW Linares OA Klein JW. Deficits in visual function after resolution of optic neuritis. Ophthalmology. (1987) 94:1029–35. doi: 10.1016/S0161-6420(87)33349-4, PMID: 3658363

[7] Rothman A Murphy OC Fitzgerald KC Button J Gordon-Lipkin E Ratchford JN. Retinal measurements predict 10-year disability in multiple sclerosis. Ann Clin Transl Neurol. (2019) 6:222–32. doi: 10.1002/acn3.674, PMID: 30847355 PMC6389740

[8] Roura E Maclair G Andorra M Juanals F Pulido-Valdeolivas I Saiz A. Cortical fractal dimension predicts disability worsening in multiple sclerosis patients. Neuroimage Clin. (2021) 30:102653. doi: 10.1016/j.nicl.2021.102653, PMID: 33838548 PMC8045041

[9] Montolio A Cegonino J Garcia-Martin E Perez Del Palomar A. Comparison of machine learning methods using Spectralis OCT for diagnosis and disability progression prognosis in multiple sclerosis. Ann Biomed Eng. (2022) 50:507–28. doi: 10.1007/s10439-022-02930-3, PMID: 35220529 PMC9001622

[10] Montolio A Cegonino J Garcia-Martin E Perez Del Palomar A. The macular retinal ganglion cell layer as a biomarker for diagnosis and prognosis in multiple sclerosis: a deep learning approach. Acta Ophthalmol. (2023). doi: 10.1111/aos.15722, PMID: 37300357

[11] Montolio A Cegonino J Orduna E Sebastian B Garcia-Martin E Perez Del Palomar A. A mathematical model to predict the evolution of retinal nerve fiber layer thinning in multiple sclerosis patients. Comput Biol Med. (2019) 111:103357. doi: 10.1016/j.compbiomed.2019.103357 31326867

[12] Berek K Hegen H Hocher J Auer M Di Pauli F Krajnc N. Retinal layer thinning as a biomarker of long-term disability progression in multiple sclerosis. Mult Scler. (2022) 28:1871–80. doi: 10.1177/13524585221097566, PMID: 35652366

[13] Tiu VE Popescu BO Enache II Tiu C Cherecheanu AP Panea CA. Serum Neurofilaments and OCT metrics predict EDSS-plus score progression in early relapse-remitting multiple sclerosis. Biomedicine. (2023) 11:606. doi: 10.3390/biomedicines11020606 PMC9953670 36831142

[14] Bsteh G Berek K Hegen H Altmann P Wurth S Auer M. Macular ganglion cell-inner plexiform layer thinning as a biomarker of disability progression in relapsing multiple sclerosis. Mult Scler. (2021) 27:684–94. doi: 10.1177/1352458520935724, PMID: 32613912

[15] Cleary PA Beck RW Anderson MM Jr Kenny DJ Backlund JY Gilbert PR. Design, methods, and conduct of the optic neuritis treatment trial. Control Clin Trials. (1993) 14:123–42. doi: 10.1016/0197-2456(93)90015-6, PMID: 8500302

[16] Beck RW Cleary PA Anderson MM Jr Keltner JL Shults WT Kaufman DI. A randomized, controlled trial of corticosteroids in the treatment of acute optic neuritis. The optic neuritis study group. N Engl J Med. (1992) 326:581–8. doi: 10.1056/NEJM199202273260901 1734247

[17] Beck RW Cleary PA Trobe JD Kaufman DI Kupersmith MJ Paty DW. The effect of corticosteroids for acute optic neuritis on the subsequent development of multiple sclerosis. The optic neuritis study group. N Engl J Med. (1993) 329:1764–9. doi: 10.1056/NEJM199312093292403, PMID: 8232485

[18] Optic Neuritis Study Group. The clinical profile of optic neuritis. Experience of the optic neuritis treatment trial.. Arch Ophthalmol. (1991) 109:1673–8. doi: 10.1001/archopht.1991.01080120057025 1841573

[19] Visual function 5 years after optic neuritis: experience of the optic neuritis treatment trial. Optic Neuritis Study Group. Arch Ophthalmol. (1997) 115:1545–52. doi: 10.1001/archopht.1997.01100160715008, PMID: 9400788

[20] Kurtzke JF. Rating neurologic impairment in multiple sclerosis: an expanded disability status scale (EDSS). Neurology. (1983) 33:1444–52. doi: 10.1212/WNL.33.11.1444 6685237

[21] Poser CM Paty DW Scheinberg L McDonald WI Davis FA Ebers GC. New diagnostic criteria for multiple sclerosis: guidelines for research protocols. Ann Neurol. (1983) 13:227–31. doi: 10.1002/ana.410130302, PMID: 6847134

[22] Luo W Deng X Xu X Song R Luo M Moss HE. Development of a prognostic model for predicting multiple sclerosis after optic neuritis: a secondary analysis of data from the optic neuritis treatment trial. J Neuroophthalmol. (2022) 42:88–96. doi: 10.1097/WNO.0000000000001424, PMID: 34860745 PMC9159903

[23] Rubin DB. Multiple imputation for nonresponse in surveys. New York: Wiley (2004). p. 258.

[24] Harrell FE Jr Califf RM Pryor DB Lee KL Rosati RA. Evaluating the yield of medical tests. JAMA. (1982) 247:2543–6. doi: 10.1001/jama.1982.03320430047030 7069920

[25] R Development Core Team. R: A language and environment for statistical computing. R Foundation for Statistical Computing: Vienna, Austria. Computing (2021).

[26] Martinez-Lapiscina EH Arnow S Wilson JA Saidha S Preiningerova JL Oberwahrenbrock T. Retinal thickness measured with optical coherence tomography and risk of disability worsening in multiple sclerosis: a cohort study. Lancet Neurol. (2016) 15:574–84. doi: 10.1016/S1474-4422(16)00068-5, PMID: 27011339

[27] Leibowitz U Alter M Halpern L. Clinical studies of multiple sclerosis in Israel. 3. Clinical course and prognosis related to age at onset. Neurology. (1964) 14:926–32. doi: 10.1212/WNL.14.10.926 14219199

[28] Leibowitz U Alter M. Clinical factors associated with increased disability in multiple sclerosis. Acta Neurol Scand. (1970) 46:53–70. doi: 10.1111/j.1600-0404.1970.tb05604.x 5412627

[29] Weinshenker BG Rice GP Noseworthy JH Carriere W Baskerville J Ebers GC. The natural history of multiple sclerosis: a geographically based study. 3. Multivariate analysis of predictive factors and models of outcome. Brain. (1991) 114:1045–56. doi: 10.1093/brain/114.2.1045, PMID: 2043940

[30] Doshi A Chataway J. Multiple sclerosis, a treatable disease. Clin Med (Lond). (2017) 17:530–6. doi: 10.7861/clinmedicine.17-6-530, PMID: 29196354 PMC6297710

[31] Lublin FD Haring DA Ganjgahi H Ocampo A Hatami F Cuklina J. How patients with multiple sclerosis acquire disability. Brain. (2022) 145:3147–61. doi: 10.1093/brain/awac016, PMID: 35104840 PMC9536294

[32] Brex PA Ciccarelli O O'Riordan JI Sailer M Thompson AJ Miller DH. A longitudinal study of abnormalities on MRI and disability from multiple sclerosis. N Engl J Med. (2002) 346:158–64. doi: 10.1056/NEJMoa011341 11796849

[33] Weinshenker BG Bass B Rice GP Noseworthy J Carriere W Baskerville J. The natural history of multiple sclerosis: a geographically based study. 2. Predictive value of the early clinical course. Brain. (1989) 112:1419–28. doi: 10.1093/brain/112.6.1419, PMID: 2597989

[34] Confavreux C Aimard G Devic M. Course and prognosis of multiple sclerosis assessed by the computerized data processing of 349 patients. Brain. (1980) 103:281–300. doi: 10.1093/brain/103.2.281, PMID: 7397479

[35] Optic Neuritis Study G. Multiple sclerosis risk after optic neuritis: final optic neuritis treatment trial follow-up. Arch Neurol. (2008) 65:727–32. doi: 10.1001/archneur.65.6.727 18541792 PMC2440583

[36] Miller DH Chard DT Ciccarelli O. Clinically isolated syndromes. Lancet Neurol. (2012) 11:157–69. doi: 10.1016/S1474-4422(11)70274-5, PMID: 22265211

[37] Bosca I Coret F Valero C Pascual AM Magraner MJ Landete L. Effect of relapses over early progression of disability in multiple sclerosis patients treated with beta-interferon. Mult Scler. (2008) 14:636–9. doi: 10.1177/1352458507086666 18566027

[38] Motamed MR Najimi N Fereshtehnejad SM. The effect of interferon-beta1a on relapses and progression of disability in patients with clinically isolated syndromes (CIS) suggestive of multiple sclerosis. Clin Neurol Neurosurg. (2007) 109:344–9. doi: 10.1016/j.clineuro.2007.01.007, PMID: 17300863

[39] Rudick RA Stuart WH Calabresi PA Confavreux C Galetta SL Radue EW. Natalizumab plus interferon beta-1a for relapsing multiple sclerosis. N Engl J Med. (2006) 354:911–23. doi: 10.1056/NEJMoa044396, PMID: 16510745

[40] Vasileiou ES Fitzgerald KC. Multiple sclerosis pathogenesis and updates in targeted therapeutic approaches. Curr Allergy Asthma Rep. (2023) 23:481–96. doi: 10.1007/s11882-023-01102-0, PMID: 37402064

[41] Palace J Bregenzer T Tremlett H Oger J Zhu F Boggild M. UK multiple sclerosis risk-sharing scheme: a new natural history dataset and an improved Markov model. BMJ Open. (2014) 4:e004073. doi: 10.1136/bmjopen-2013-004073, PMID: 24441054 PMC3902459

[42] Swanton JK Fernando KT Dalton CM Miszkiel KA Altmann DR Plant GT. Early MRI in optic neuritis: the risk for disability. Neurology. (2009) 72:542–50. doi: 10.1212/01.wnl.0000341935.41852.82 19204264

[43] Fisniku LK Brex PA Altmann DR Miszkiel KA Benton CE Lanyon R. Disability and T2 MRI lesions: a 20-year follow-up of patients with relapse onset of multiple sclerosis. Brain. (2008) 131:808–17. doi: 10.1093/brain/awm329 18234696

[44] Beck RW Smith CH Gal RL Xing D Bhatti MT Brodsky MC. Neurologic impairment 10 years after optic neuritis. Arch Neurol. (2004) 61:1386–9. doi: 10.1001/archneur.61.9.1386, PMID: 15364684

[45] Chen JJ Tobin WO Majed M Jitprapaikulsan J Fryer JP Leavitt JA. Prevalence of myelin oligodendrocyte glycoprotein and Aquaporin-4-IgG in patients in the optic neuritis treatment trial. JAMA Ophthalmol. (2018) 136:419–22. doi: 10.1001/jamaophthalmol.2017.6757, PMID: 29470571 PMC5876803

